# Supplementary material for: A Humanized Yeast Phenomic Model of Deoxycytidine Kinase to Predict Genetic Buffering of Nucleoside Analog Cytotoxicity
Source: Genes (Basel). 2019 Sep 30;10(10):770. doi: 10.3390/genes10100770 (PMC6826991; doi:10.3390/genes10100770)
Supplement: Supplementary file 1 [file genes-10-00770-s001.zip › Additional_Files/Additional_File7_GOTermHeatmaps.zip!/Additional_File7_GOTermHeatmaps/large_ribosomal_subunit.pdf]

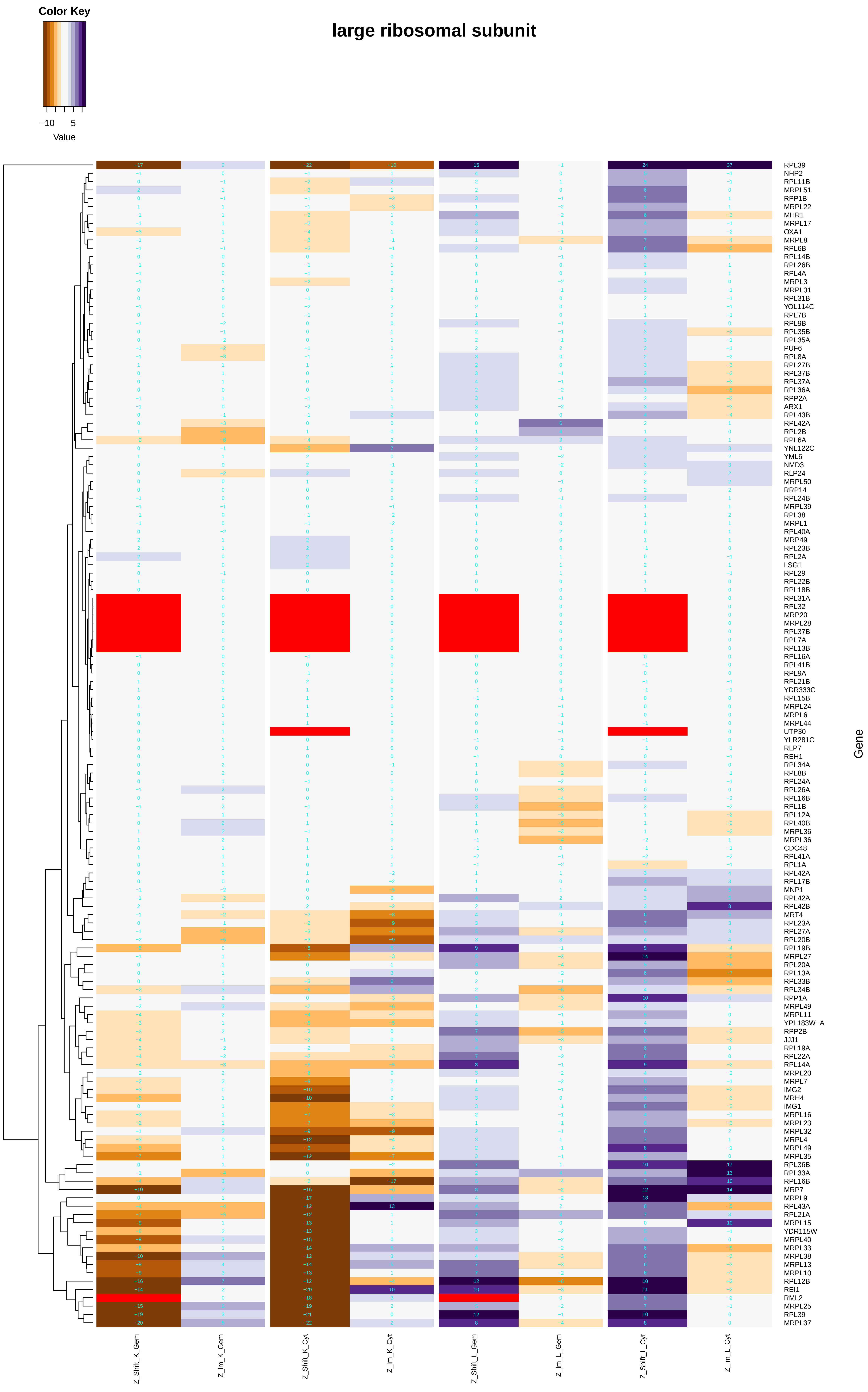
large ribosomal subunit
Color Key
−10
5
Value
RPL39
NHP2
RPL11B
MRPL51
RPP1B
MRPL22
MHR1
MRPL17
OXA1
MRPL8
RPL6B
RPL14B
RPL26B
RPL4A
MRPL3
MRPL31
RPL31B
YOL114C
RPL7B
RPL9B
RPL35B
RPL35A
PUF6
RPL8A
RPL27B
RPL37B
RPL37A
RPL36A
RPP2A
ARX1
RPL43B
RPL42A
RPL2B
RPL6A
YNL122C
YML6
NMD3
RLP24
MRPL50
RRP14
RPL24B
MRPL39
RPL38
MRPL1
RPL40A
MRP49
RPL23B
RPL2A
LSG1
RPL29
RPL22B
RPL18B
RPL31A
RPL32
MRP20
MRPL28
RPL37B
RPL7A
RPL13B
RPL16A
RPL41B
RPL9A
RPL21B
YDR333C
RPL15B
MRPL24
MRPL6
MRPL44
UTP30
YLR281C
RLP7
REH1
RPL34A
RPL8B
RPL24A
RPL26A
RPL16B
RPL1B
RPL12A
RPL40B
MRPL36
MRPL36
CDC48
RPL41A
RPL1A
RPL42A
RPL17B
MNP1
RPL42A
RPL42B
MRT4
RPL23A
RPL27A
RPL20B
RPL19B
MRPL27
RPL20A
RPL13A
RPL33B
RPL34B
RPP1A
MRPL49
MRPL11
YPL183W−A
RPP2B
JJJ1
RPL19A
RPL22A
RPL14A
MRPL20
MRPL7
IMG2
MRH4
IMG1
MRPL16
MRPL23
MRPL32
MRPL4
MRPL49
MRPL35
RPL36B
RPL33A
RPL16B
MRP7
MRPL9
RPL43A
RPL21A
MRPL15
YDR115W
MRPL40
MRPL33
MRPL38
MRPL13
MRPL10
RPL12B
REI1
RML2
MRPL25
RPL39
MRPL37
Gene
Z_Shift_K_Gem
Z_Im_K_Gem
Z_Shift_K_Cyt
Z_Im_K_Cyt
Z_Shift_L_Gem
Z_Im_L_Gem
Z_Shift_L_Cyt
Z_Im_L_Cyt

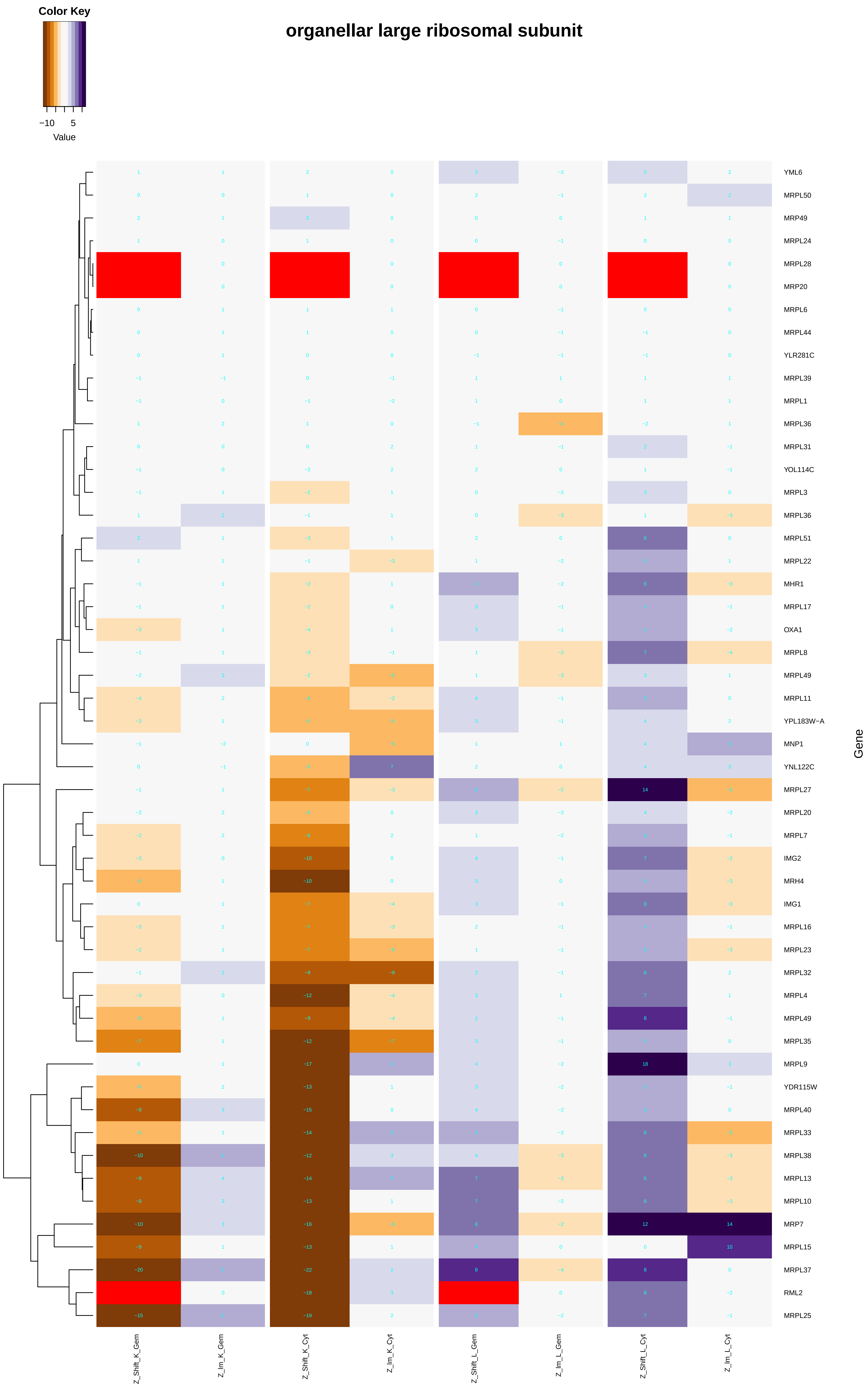
organellar large ribosomal subunit
Color Key
−10
5
Value
Gene
YML6
MRPL50
MRP49
MRPL24
MRPL28
MRP20
MRPL6
MRPL44
YLR281C
MRPL39
MRPL1
MRPL36
MRPL31
YOL114C
MRPL3
MRPL36
MRPL51
MRPL22
MHR1
MRPL17
OXA1
MRPL8
MRPL49
MRPL11
YPL183W−A
MNP1
YNL122C
MRPL27
MRPL20
MRPL7
IMG2
MRH4
IMG1
MRPL16
MRPL23
MRPL32
MRPL4
MRPL49
MRPL35
MRPL9
YDR115W
MRPL40
MRPL33
MRPL38
MRPL13
MRPL10
MRP7
MRPL15
MRPL37
RML2
MRPL25
Z_Shift_K_Gem
Z_Im_K_Gem
Z_Shift_K_Cyt
Z_Im_K_Cyt
Z_Shift_L_Gem
Z_Im_L_Gem
Z_Shift_L_Cyt
Z_Im_L_Cyt

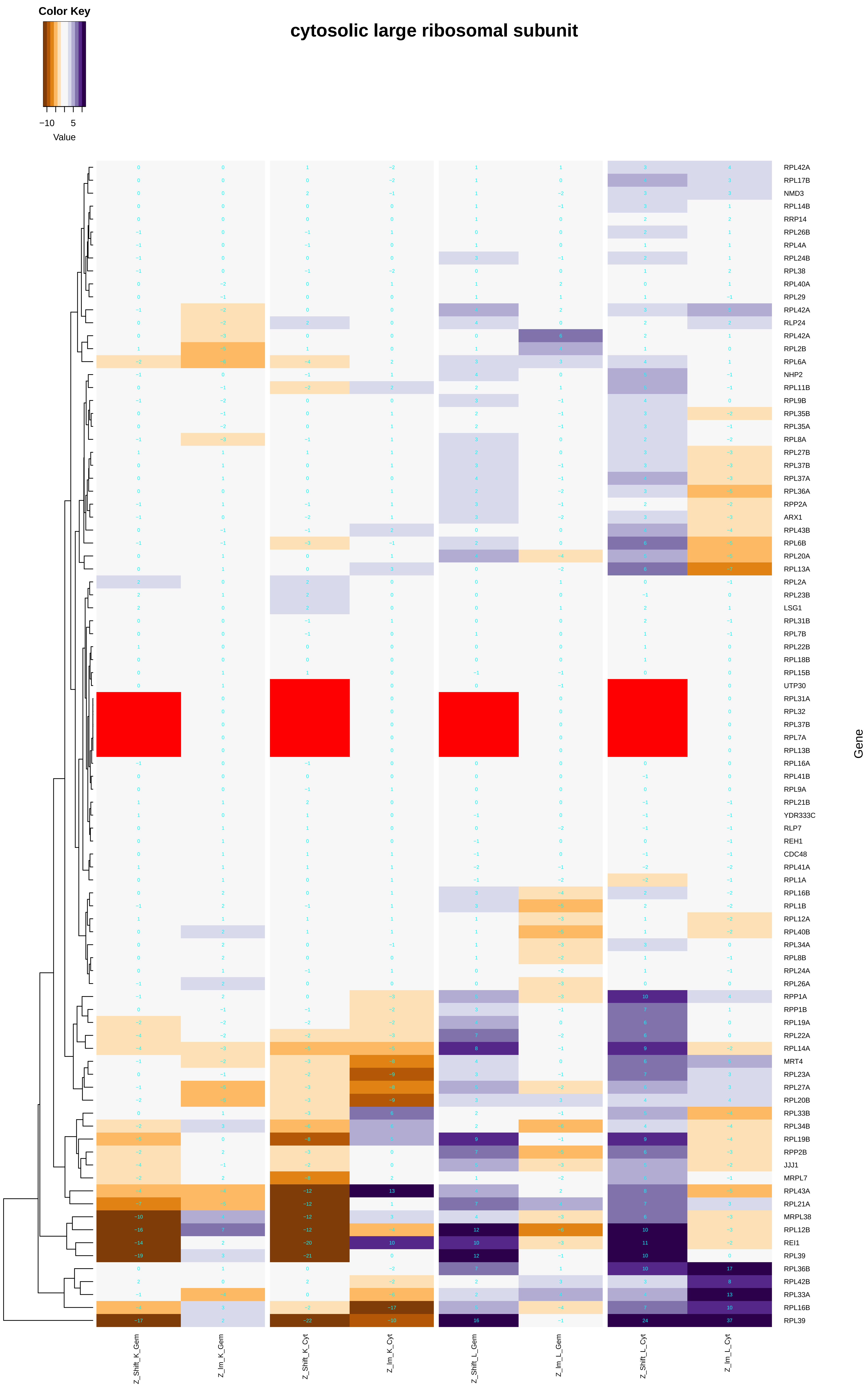
cytosolic large ribosomal subunit
Color Key
−10
5
Value
RPL42A
RPL17B
NMD3
RPL14B
RRP14
RPL26B
RPL4A
RPL24B
RPL38
RPL40A
RPL29
RPL42A
RLP24
RPL42A
RPL2B
RPL6A
NHP2
RPL11B
RPL9B
RPL35B
RPL35A
RPL8A
RPL27B
RPL37B
RPL37A
RPL36A
RPP2A
ARX1
RPL43B
RPL6B
RPL20A
RPL13A
RPL2A
RPL23B
LSG1
RPL31B
RPL7B
RPL22B
RPL18B
RPL15B
UTP30
RPL31A
RPL32
RPL37B
RPL7A
RPL13B
RPL16A
RPL41B
RPL9A
RPL21B
YDR333C
RLP7
REH1
CDC48
RPL41A
RPL1A
RPL16B
RPL1B
RPL12A
RPL40B
RPL34A
RPL8B
RPL24A
RPL26A
RPP1A
RPP1B
RPL19A
RPL22A
RPL14A
MRT4
RPL23A
RPL27A
RPL20B
RPL33B
RPL34B
RPL19B
RPP2B
JJJ1
MRPL7
RPL43A
RPL21A
MRPL38
RPL12B
REI1
RPL39
RPL36B
RPL42B
RPL33A
RPL16B
RPL39
Gene
Z_Shift_K_Gem
Z_Im_K_Gem
Z_Shift_K_Cyt
Z_Im_K_Cyt
Z_Shift_L_Gem
Z_Im_L_Gem
Z_Shift_L_Cyt
Z_Im_L_Cyt

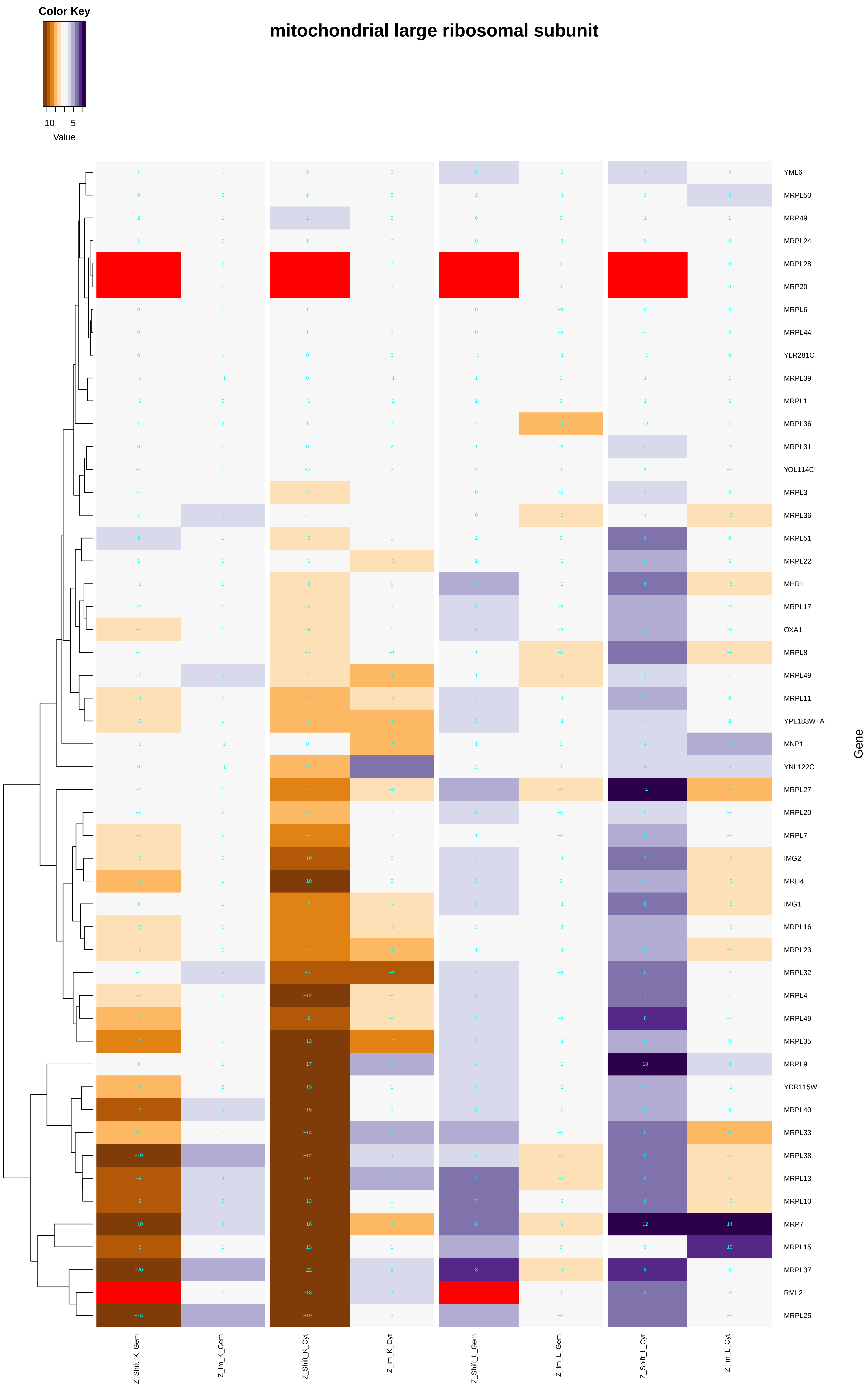
Color Key
−10
5
Value
mitochondrial large ribosomal subunit
YML6
MRPL50
MRP49
MRPL24
MRPL28
MRP20
MRPL6
MRPL44
YLR281C
MRPL39
MRPL1
MRPL36
MRPL31
YOL114C
MRPL3
MRPL36
MRPL51
MRPL22
MHR1
MRPL17
OXA1
MRPL8
MRPL49
MRPL11
YPL183W−A
MNP1
YNL122C
MRPL27
MRPL20
MRPL7
IMG2
MRH4
IMG1
MRPL16
MRPL23
MRPL32
MRPL4
MRPL49
MRPL35
MRPL9
YDR115W
MRPL40
MRPL33
MRPL38
MRPL13
MRPL10
MRP7
MRPL15
MRPL37
RML2
MRPL25
Gene
Z_Shift_K_Gem
Z_Im_K_Gem
Z_Shift_K_Cyt
Z_Im_K_Cyt
Z_Shift_L_Gem
Z_Im_L_Gem
Z_Shift_L_Cyt
Z_Im_L_Cyt
